# The gut microbiome in colorectal cancer: mechanisms of carcinogenesis and emerging microbiota-targeted therapies

**DOI:** 10.1007/s12672-025-04367-1

**Published:** 2026-01-07

**Authors:** Yue Li, Xingyu Shen, Deqiang Wang, Kang Sun

**Affiliations:** 1 Department of Gastrointestinal surgery, Affiliated Hospital of Jiangsu University, 438 Jiefang Road, Zhenjiang, 212000 China; 2 Department of Oncology, Affiliated Hospital of Jiangsu University, 438 Jiefang Road, Zhenjiang, 212000 China

**Keywords:** Colorectal cancer, Microbiome, Carcinogenesis, Chemoresistance, Fecal microbial transplantation

## Abstract

Colorectal cancer (CRC) remains a leading cause of cancer-related mortality globally. Beyond established genetic and environmental risk factors, the gut microbiome is now recognized as a pivotal contributor to CRC pathogenesis, progression, and therapeutic response. This review synthesizes current evidence on how dysbiosis and specific pathogenic bacteria—notably *Fusobacterium nucleatum* (Fn), *Enterotoxigenic Bacteroides fragilis* (ETBF), and *Escherichia coli* carrying the polyketide synthase genomic island (*pks*^+^* E. coli*)—may drive carcinogenesis through chronic inflammation, genotoxic metabolite production, immune evasion, and epigenetic reprogramming. Critically, we explore the microbiome’s dual role in modulating conventional therapies: Fn is linked to chemotherapy resistance and metastasis, while certain commensals may enhance radiotherapy and immunotherapy efficacy. We further evaluate emerging microbiota-targeted strategies, including fecal microbiota tra nsplantation (FMT), probiotics, prebiotics, postbiotics and precision antibiotics, which hold promise for restoring microbial balance and overcoming treatment resistance. By integrating mechanistic insights with clinical evidence, this review provides a foundation for leveraging the microbiome in CRC diagnosis, prognosis, and next-generation therapeutic approaches.

## Introduction

CRC ranks as the second leading cause of cancer-related mortality and third most commonly diagnosed malignancy worldwide, with over 1.9 million new cases annually. Despite advances in screening and therapeutic interventions, CRC accounted for approximately 930,000 deaths in 2020, representing 10% of all cancer mortality globally [1], underscoring the urgent need for novel treatment strategies. While established risk factors -including smoking, dietary habits, obesity, inflammatory bowel disease, and genetic mutations-have been extensively characterized [2–4], mounting evidence implicates gut microbiota as crucial players in CRC pathogenesis and progression.

The intestinal microbiota comprises an estimated 4 × 10¹³ bacterial cells, approximately equal in number to human cells and collectively weighing around 0.2 kg [5]. Under normal physiological conditions, these commensal microbes collaborate to maintain a balanced intestinal ecosystem by facilitating digestion, engaging in metabolic processes, modulating the immune system, and supporting epithelial cell maturation [6]. Dominant genera such as *Bacteroides* and *Bifidobacterium* typically maintain a symbiotic relationship with the host [7]. However, microbial dysbiosis—characterized by the depletion of commensal taxa (e.g., *Lactobacillus*) and overgrowth of pathobionts (e.g., *Fusobacterium nucleatum*)—has been linked to CRC through multiple mechanisms: chronic inflammation, genotoxic metabolite production, and immune evasion.

Recent advances in metagenomic sequencing have identified specific CRC-associated bacteria, such as *pks*^+^* E. coli* and ETBF [8], which directly damage host DNA or modulate oncogenic signaling pathways (e.g., Wnt/β-catenin) [9, 10]. Intriguingly, certain microbial strains (e.g., *Lactobacillus reuteri*) may exert protective effects [11], underscoring the microbiota’s dual role in CRC. To translate these insights into clinical practice, two critical and specific research questions must be addressed: first, what are the temporal dynamics of microbial changes during CRC progression, from initial adenoma to invasive carcinoma and metastasis? Second, which patient populations—defined by factors such as genetic predisposition, specific microbial signatures, or disease stage—would benefit most from targeted microbiota-based interventions? Clarifying these aspects is essential for developing precise, microbiome-informed strategies for CRC prevention, diagnosis, and therapy.

This review synthesizes current knowledge from preclinical and clinical studies published up to December 2025. We conducted a literature search using PubMed and Web of Science databases with keywords including ‘colorectal cancer,’ ‘gut microbiome,’ ‘*Fusobacterium nucleatum*,’ ‘*enterotoxigenic Bacteroides fragilis*,’ ‘*colibactin*,’ ‘immunotherapy,’ ‘Probiotics,*’* and‘fecal microbiota transplantation.’ Emphasis was placed on recent primary research, mechanistic studies, and clinical trials to provide a comprehensive and updated perspective. The review is structured along three axes: (1) mechanistic insights into microbial carcinogenesis (2), the translational potential of microbiota-based diagnostics and therapeutics, and (3) challenges, contextual factors, and future directions. By integrating this evidence, we aim to provide a roadmap for harnessing the microbiome to improve CRC management.

## Mechanisms of microbiota-driven colorectal carcinogenesis

The gut microbiota critically shapes CRC progression through multifaceted mechanisms, principally involving immune modulation, chronic inflammatory responses, and epigenetic reprogramming.

### Remodeling of the immune microenvironment

Remodeling of the immune microenvironment The gut microbiota primes a pro-tumorigenic immune response through pathogen-associated molecular patterns, with distinct individual bacterial species driving unique immunomodulatory pathways. *Fusobacterium nucleatum* (Fn): Fn-derived lipopolysaccharide (LPS) triggers tumor-associated macrophages (TAMs) to produce interleukin-6(IL-6), which activates STAT3 signaling to promote epithelial cell proliferation [12]. Additionally, Fn-infected CRC cells secrete chemokines (CCL2, CXCL1) that recruit CCR2⁺ monocytes and CXCR2⁺ granulocytic myeloid-derived suppressor cells, hereby forming an immunosuppressive microenvironment [13].

**Fig. 1:**
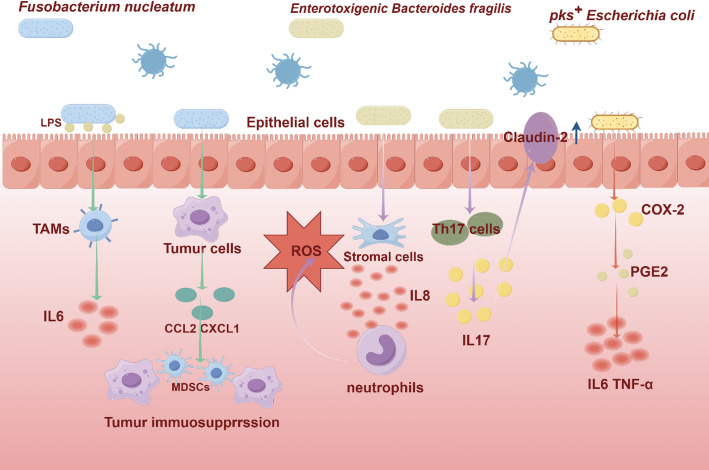
This diagram illustrates the interaction between specific pathogenic bacteria (such as *Fusobacterium nucleatum*, *Enterotoxigenic Bacteroides fragilis* and *pks*^+^*Escherichia coli*) and host colon epithelial cells and immune cells. The key processes described include: (1) disruption of epithelial barrier integrity and adhesion; (2) Activate pro-inflammatory and carcinogenic signaling pathways; (3) Recruitment and polarization of immune cells (such as Th17 cells, myeloid derived suppressor cells, tumor associated macrophages) to form an immunosuppressive tumor microenvironment; Arrows indicate activation, induction, or recruitment; CRC, colorectal cancer; MDSCs, myeloid-derived suppressor cells; TAMs, tumor-associated macrophages; LPS, lipopolysaccharide; IL-16, interleukin-16; IL-8, interleukin-8; IL-17, interleukin-17; COX-2, cyclooxygenase-2;PGE₂, prostaglandin E₂; Th17, T helper 17. ROS, reactive oxygen species.CCL2, C-C motif chemokine ligand 2; CXCL1, C-X-C motif chemokine ligand 1; TNFα, Tumor Necrosis Factor-α. Figures were made with Figdraw

*Enterotoxigenic Bacteroides fragilis* (ETBF): ETBF drives T helper 17 (Th17) cell differentiation, leading to elevated IL-17 levels; this is associated with dysregulated upregulation of the pore-forming tight junction protein claudin-2, which increases intestinal permeability [14]. ETBF-derived metalloproteases also stimulate stromal cells to release IL-8, recruiting neutrophils that further amplify inflammation via reactive oxygen species (ROS) production [15]. *pks*^+^* E. coli*: This bacterium amplifies inflammatory signaling through COX-2/PGE₂ pathways [16]. Furthermore, *pks*^+^* E. coli* infection is associated with the depletion of CD3⁺ and CD8⁺ T cells within tumors, while concurrently recruiting or activating myeloid-derived suppressor cells and M2-like tumor-associated macrophages, thereby establishing a potent barrier to anti-tumor immunity(Figure 1).

These distinct immunomodulatory events collectively contribute to a pro-tumorigenic milieu. Their influence may also vary temporally: for instance, early ETBF colonization may initiate a Th17-driven chronic inflammatory state conducive to tumor initiation, while later enrichment of Fn in established tumors is more strongly linked to recruiting immunosuppressive myeloid cells that facilitate progression and metastasis.

While immune microenvironment remodeling creates favorable conditions for tumor growth, direct genotoxic insults from specific microbiota represent another key carcinogenic pathway. These two mechanisms often act synergistically: immune dysregulation-induced chronic inflammation increases epithelial permeability, allowing genotoxin-producing bacteria to directly interact with colonic epithelial cells and initiate malignant transformation.

### Genotoxicity and epigenetic regulation

The intestinal microbiome exerts a significant influence on CRC through a variety of mechanisms, including epigenetic regulation. Microbial-derived metabolites, particularly *short-chain fatty acids* (SCFAs), play a crucial role in this process. These metabolites mediate programmed cell death in neoplastic cells via G protein-coupled receptor signaling pathways, thereby regulating gene transcription and inducing metabolic alterations in vivo [17]. Furthermore, intestinal microorganisms modulate epigenetic regulation via DNA methylation processes, a mechanism that may offer therapeutic potential for CRC management [18].

Beyond SCFA-mediated histone deacetylase (HDAC) inhibition, gut microbes influence broader epigenetic landscapes that contribute to CRC pathogenesis. Notably, histone ubiquitination, a key post-translational modification, regulates chromatin function. Its dysregulation can lead to a chromatin “re-wiring” characterized by increased accessibility at promoters and enhancers rich in pioneer transcription factor motifs. This altered transcriptional landscape may facilitate the upregulation of pro-tumorigenic genes [19]. Concurrently, histone lactylation has emerged as a novel and significant modification in CRC, with its elevation is associated with poor prognosis in patients, underscoring its clinical relevance despite the complexity of its functional roles in tumor biology [20].

Epigenetic dysregulation often converges with genetic alterations to fully transform colorectal epithelium. Mutations in tumor suppressor genes represent a core event in CRC initiation, and a complex interplay exists between the microbial ecosystem and these critical genetic nodes. Beyond direct metabolite-mediated effects, gut microbes may also influence the stability of key CRC driver genes through direct or indirect interactions with the host genome. In recent years, the association between *Fusobacterium* species and genetic alterations in CRC has garnered increasing attention. For example, enrichment of *Fusobacterium mortiferum* in patients with colorectal polyps has been statistically associated with *APC* gene mutation status [21] suggesting that different *Fusobacterium* species may commonly be linked to abnormalities in critical host tumor suppressor genes.Among these, Fn, as the most extensively studied oncogenic bacterium, has had its associated mechanisms more clearly elucidated. Research indicates that Fn can adhere directly to epithelial cells via its virulence factor FadA, activating the Wnt/β-catenin signaling pathway [22]. Notably, this pathway is the core downstream pathway following *APC* gene inactivation. Therefore, Fn infection may synergize with *APC* loss to jointly drive genomic instability and tumorigenesis.

The tumor suppressor p53 serves as another pivotal interface between microbial signals and intestinal transformation. Its function is deeply and bidirectionally intertwined with the gut microbiota [23], with outcomes that depend critically on cellular context. Under physiological or mildly inflammatory conditions, a normal gut microbiota helps maintain the tumor-suppressive activity of p53. For instance, a healthy microbiota can suppress the expression of the oncogenic long non-coding RNA *SNHG9*, thereby preventing *SNHG9* from interfering with *SIRT1*—a crucial deacetylase for p53—thus preserving p53-dependent transcription and its capacity to induce cell cycle arrest or apoptosis [24]. However, when the p53 gene itself is mutated, the presence of the gut microbiota may instead drive tumor progression. Direct evidence from studies using the CKIa^Δgut^ p53 ^R172H^ mouse model demonstrates that the loss of WNT pathway suppression by this specific p53 mutant in the distal gut is entirely dependent on the presence of gut microbiota. Upon microbiota eradication, the intestinal dysplasia in mutant mice was alleviated, accompanied by reduced WNT signaling and decreased cell proliferation [25]. This reveals that the microbiota can antagonize the residual tumor-suppressive functions of mutant p53, even converting it into a pro-oncogenic factor.

In summary, this section illustrates how the gut microbiome contributes to colorectal carcinogenesis through a multi-layered interplay with host regulatory systems. It not only reshapes the epigenetic landscape via metabolites and chromatin modifications but also directly engages with core tumor suppressor pathways—exemplified by *APC* and p53. These interactions range from synergistic cooperation with genetic alterations to context-dependent functional switching of key gatekeepers, highlighting the microbiota as a dynamic and integral component of the tumor microenvironment that influences both the initiation and progression of CRC.

## Pathogenic bacteria associated with colorectal cancer

### *Fusobacterium nucleatum*

*Fusobacterium* spp., an anaerobic bacterial genus, has been increasingly recognized as a key microbial contributor to colorectal carcinogenesis. Metagenomic analyses consistently demonstrate significant enrichment of this genus in both fecal samples and tumor tissues of CRC patients, with particularly strong associations observed in advanced disease stages [26]. Elevated burden of *Fusobacterium* correlates with multiple poor prognostic indicators, including reduced overall survival, chemotherapy resistance, and heightened metastatic potential [27, 28]. Notably, comparative genomic studies further reveal that within this genus, the species *Fusobacterium nucleatum*, especially its subspecies animalis (often termed *Fusobacterium nucleatum animalis* or Fna), is predominantly enriched in the CRC tumor microenvironment. Importantly, Fna is not a homogeneous entity but comprises two major clades: Fna C1 and Fna C2. While Fna C1 remains largely confined to the oral cavity, Fna C2 is the clade specifically and significantly enriched in colorectal tumor tissues [29]. This CRC-associated clade harbors enhanced genetic adaptations for metabolizing gut-derived nutrients and resisting pH stress, which likely underlies its successful colonization and persistence in the gastrointestinal tumor niche. Clinically, the presence of Fna C2 shows higher specificity and positive predictive value for CRC than broader taxonomic assignments and correlates with key molecular features such as mismatch repair status and HER2 expression [30], suggesting its potential role in shaping disease progression and the prospect of precise intervention for specific carcinogenic strains in the future.

**Fig. 2:**
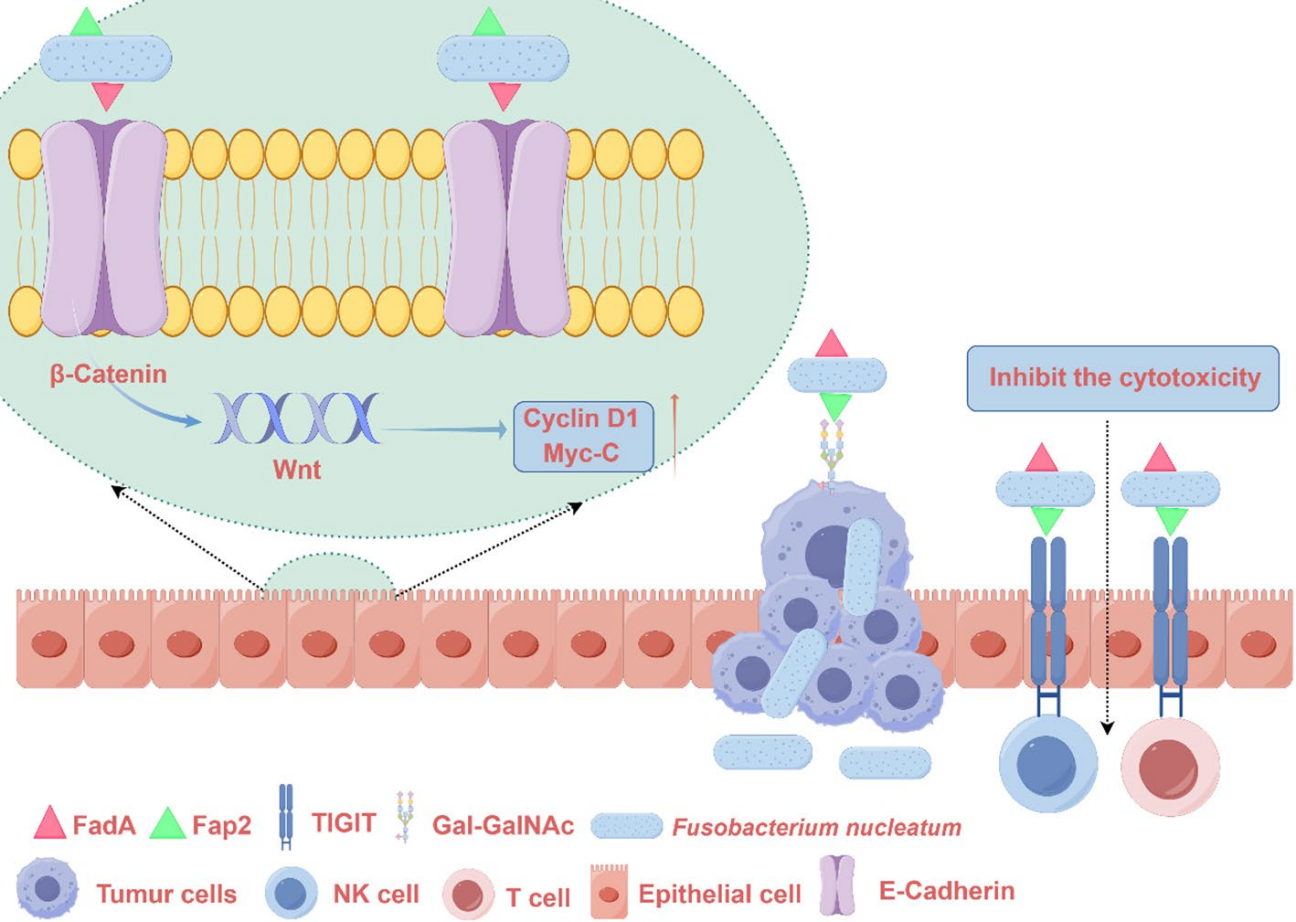
The diagram highlights two major virulence factors of Fn: FadA and Fap2. Left panel (FadA-mediated effects): FadA binding to E-cadherin disrupts intercellular adhesion and activates the β-catenin signaling pathway, leading to the upregulation of target genes (e.g., Cyclin D1, Myc-C) that promote proliferation. Right panel (Fap2-mediated effects): Fap2 mediates specific enrichment of Fn in CRC tissues by binding to tumor-associated Gal-GalNAc glycans. Furthermore, Fap2 binding to the inhibitory receptor TIGIT on natural killer cells suppresses their cytotoxic function, facilitating immune evasion. Collectively, these mechanisms promote tumor growth, invasion, and the establishment of an immunosuppressive niche. Abbreviations: FadA, Fusobacterium adhesion A; Fap2, outer-membrane adhesin; Gal-GalNAc, D-galactose-β [1–3]-N-acetyl-D-galactosamine; TIGIT, T-cell immunoreceptor with Ig and ITIM domains; NK cell, natural killer cell. Figures were made with Figdraw

The pathogenic effects of Fn are primarily mediated through two well-characterized virulence factors: the FadA adhesin and the outer-membrane adhesin Fap2. As the predominant virulence determinant, FadA facilitates robust host cell binding while compromising epithelial and endothelial integrity. This dual action enables Fn to preferentially colonize CRC tissues, where it disrupts intercellular tight junctions and promotes tumor cell invasion and metastasis. At the molecular level, FadA triggers E-cadherin/β-catenin signaling pathway activation, resulting in Chk2 overexpression that drives both DNA damage and enhanced cellular proliferation [31, 32] (Fig. 2).

Beyond driving epithelial proliferation, Fn also profoundly reshapes the tumor immune microenvironment. On one hand, its components, such as lipopolysaccharide (LPS), can activate pattern recognition receptors (e.g., Toll-like receptors) on host immune and epithelial cells, leading to the robust production of pro-inflammatory cytokines like IL-6, IL-8, and TNF-α. This creates a chronic inflammatory milieu that favors tumor cell survival and progression. On the other hand, Fn employs the adhesin Fap2 for more specific immunosuppression. Seminal work by Abed et al. [33] revealed that Fap2 specifically recognizes the tumor-associated glycan D-galactose-β (1-3)-N-acetyl-D-galactosamine (Gal-GalNAc), enabling selective enrichment of Fn within CRC tissues [34]. This specific molecular interaction creates a self-reinforcing tumor niche that facilitates persistent bacterial colonization. Importantly, therapeutic targeting of this axis appears feasible, as enzymatic removal of O-glycans—which carry Gal-GalNAc moieties—significantly impairs Fn tumor tropism. Beyond mediating bacterial adhesion, Fap2 plays a pivotal immunomodulatory role through its interaction with the inhibitory receptor TIGIT on natural killer (NK) cells [35]. This interaction initiates immunosuppressive signaling that functionally impairs NK cell cytotoxicity, effectively disabling a critical arm of anti-tumor immunity. The resultant immunosuppressive microenvironment not only facilitates Fn persistence but also promotes tumor immune evasion and progression (Fig. 2).

Fn infection is linked to specific alterations in host miRNA expression profiles, which may influence multiple hallmarks of cancer. Current evidence associates Fn infection with differential miRNA expression that correlates with oncogenic processes such as cellular proliferation, metastatic dissemination, and epithelial-mesenchymal transition (EMT) [36, 37].

At the mechanistic level, Fn engages TLR4 signaling to upregulate oncogenic miR-21, which in turn silences the tumor suppressor RASA1, contributing to uncontrolled cell proliferation [38]. Furthermore, Fn infection is associated with the suppression of exosomal miR-122-5p via the FUT8/TGF-β1/Smads axis, a change linked to enhanced EMT and invasive behavior [39]. Notably, miR-31 upregulation has been implicated in Fn-driven tumorigenesis in murine models, as its genetic deletion conferred a tumor-resistant phenotype [40]. Fn infection also contributes to microenvironmental reprogramming. Through CCL20-dependent macrophage recruitment and NF-κB activation, Fn infection is associated with suppression of miR-1322, which may facilitate metastatic progression [41].

These observations highlight the translational potential of miRNA signatures as diagnostic and prognostic biomarkers in CRC progression. The identification of distinct miRNA expression profiles associated with Fn infection could facilitate the development of novel stratification strategies for CRC management. Nevertheless, the molecular mechanisms by which Fn infection drives miRNA dysregulation remain incompletely characterized. Comprehensive investigations are needed to delineate the bacterial-epigenetic interactions, host-microbe signaling pathways, and their integration with tumor microenvironment dynamics. Such studies are necessary to fully understand the role of Fn in colorectal carcinogenesis.

### Enterotoxigenic bacteroides fragilis

The virulence of *Bacteroides fragilis* is fundamentally governed by strain-specific genetic determinants, particularly the presence of a pathogenicity island encoding critical virulence factors and biofilm formation capacity. The key virulence factor is the secreted Bacteroides fragilis toxin (Bft), a zinc-dependent metalloprotease. These genomic differences segregate *Bacteroides fragilis* into two functionally distinct subtypes: the *Non-Toxigenic Bacteroides fragilis* (NTBF), *Enterotoxigenic Bacteroides fragilis* (ETBF) [42]. The well-characterized Bft toxin is central to ETBF pathogenesis. Its primary molecular mechanism involves the specific proteolytic cleavage of the extracellular domain of E-cadherin on colonic epithelial cells. This cleavage serves two critical oncogenic functions: first, it directly compromises epithelial barrier integrity, increasing permeability; second, and more significantly, the cleaved E-cadherin fragment initiates intracellular signaling that leads to the sustained nuclear translocation and activation of β-catenin. The consequent dysregulation of Wnt/β-catenin target genes, such as c-Myc and Cyclin D1, drives excessive cellular proliferation and represents a direct molecular link between ETBF colonization and neoplastic transformation.

In assessing the association between *Bacteroides fragilis* and colorectal carcinogenesis, distinguishing ETBF from NTBF is crucial. Current diagnostic approaches primarily rely on detecting the *bft* gene via PCR or metagenomic sequencing. However, several diagnostic challenges persist: the presence of the *bft* gene does not invariably equate to the production of biologically active toxin, and mixed colonization of ETBF and NTBF is common within individual patients. These factors can compromise the specificity and quantitative accuracy of detection, underscoring the need for functional assays to confirm toxin activity in future studies for more precise risk stratification.

ETBF colonization establishes a pro-tumorigenic cascade through sustained STAT3 activation, creating a chronic inflammatory milieu that drives malignant transformation. Central to this process are two key cytokine pathways: IL-17-mediated chronic inflammation and IL-8-dependent immune cell recruitment [43, 44]. IL-17 not only perpetuates tissue inflammation but also directly promotes tumor cell survival and proliferation through multiple oncogenic pathways. Concurrently, the IL-8/STAT3 axis forms a self-reinforcing inflammatory loop that recruits immunosuppressive cell populations, effectively remodeling the tumor microenvironment to favor cancer progression [45].

Beyond its soluble toxin-mediated actions, the capacity of ETBF to form biofilms critically amplifies its oncogenic potential. These biofilms are not passive barriers but dynamic pathological units. Their detection in colorectal cancer tissues and adjacent mucosa suggests persistent, long-term interaction with the epithelium [46]. This sustained biofilm presence leads to recurrent mucosal injury, chronic inflammation, and compensatory epithelial proliferation. Crucially, the biofilm’s protective architecture facilitates immune evasion, allowing constituent bacteria like ETBF to continuously deliver toxins and carcinogenic metabolites, thereby establishing and maintaining a tumor-permissive microenvironment.

Beyond its inflammatory effects, ETBF employs two direct mechanisms of oncogenesis: First, through induction of oxidative stress via intracellular ROS accumulation, and second, via proteolytic degradation of E-cadherin—a critical component of epithelial cell junctions [47]. As detailed above, this Bft-mediated E-cadherin degradation is a trigger for pro-proliferative signaling. The loss of E-cadherin function disrupts epithelial barrier integrity, increasing cellular permeability and facilitating tumor cell invasion and metastasis [48]. Together, these mechanisms create a permissive niche characterized by genomic instability, dysfunctional cellular signaling, and microenvironmental alterations that collectively drive tumor initiation and progression.

Mounting evidence highlights the crucial role of ETBF in colorectal carcinogenesis through multiple molecular pathways. Liu et al. [49] elucidated a novel TLR4-dependent mechanism wherein ETBF infection activates nuclear factor of activated T cells 5, leading to upregulated expression of histone demethylase JMJD2B. This epigenetic reprogramming promotes cancer stem cell-like properties in colorectal epithelial cells, potentially driving tumor initiation and progression. These findings were further corroborated in subsequent animal studies demonstrating ETBF’s tumorigenic potential in experimental CRC models.

Clinical investigations by Zamani et al. [50] strengthened the ETBF-CRC connection by identifying significant associations between specific *B. fragilis* virulence genes and early-stage colorectal neoplasia. Importantly, ETBF has been consistently detected in mucosal biopsies from patients with precancerous lesions and low-grade dysplasia, with colonization rates correlating with disease severity. These collective findings position ETBF as both: (1) a promising biomarker for early CRC detection, particularly in high-risk populations, and (2) a potential therapeutic target for intercepting the adenoma-carcinoma sequence.

### pks^+^* Escherichia coli*

*Escherichia coli*, a ubiquitous commensal in the human gastrointestinal microbiota, exhibits strain-dependent pathogenic potential through specific genomic adaptations. Notably, a subset of *E. coli* strains within the polyketide synthase genomic island B2 group contains a 54 kb biosynthetic gene cluster encoding the genotoxin colibactin. This microbial secondary metabolite exerts potent carcinogenic effects by inducing DNA interstrand crosslinks, resulting in double-strand breaks and subsequent genomic instability that drives malignant transformation [51, 52].

Colibactin-producing *Escherichia coli* (CoPEC) is associated with an increased prevalence of CRC [53], with CoPEC infection fostering a pro-carcinogenic immune environment by depleting CD3^+^ and CD8^+^ T cells, thereby potentially enhancing tumor resistance to immunotherapy [54]. Additionally, CoPEC activates an inflammatory response in colorectal epithelial cells via lipopolysaccharide binding to TLR4, further contributing to tumor progression [55]. Moreover, CoPEC infection significantly upregulates COX-2 expression in macrophages, thereby enhancing their pro-tumor activity [56].

The precise contribution of CoPEC to CRC is complex and appears to be influenced by multiple contextual factors, leading to seemingly conflicting reports in the literature regarding its association with cancer stage and risk.

#### Stage-dependence and critical appraisal of conflicting evidence

The association between *pks*^+^* E. coli* and colorectal cancer is not monolithic but exhibits significant stage-dependency and geographical heterogeneity, necessitating a critical, systematic evaluation of the conflicting literature.

#### Synthesis of stage-dependent evidence

A growing body of evidence suggests that the role of *pks*^+^* E. coli* may shift across the CRC continuum, explaining some of the apparent contradictions in reported correlations:

Initiation phase (Adenoma/Early Carcinoma): The strongest mechanistic case exists for its role in tumor initiation. The genotoxin colibactin directly induces double-strand DNA breaks and leaves a characteristic mutational signature in colonic epithelial cells, a finding robustly replicated in organoid and murine models [57]. This aligns with clinical observations of *pks*^+^* E. coli* enrichment in patients with colorectal adenomas or very early-stage (Stage I) cancers, where it may act as a direct genomic instigator.

Progression phase: In established tumors, the direct genotoxic role may become less dominant. Here, *pks*^+^* E. coli* contributes to tumor progression and therapy resistance through indirect mechanisms: shaping an immunosuppressive tumor microenvironment, promoting stemness and epithelial-mesenchymal transition, and sustaining chronic inflammation via COX-2/PGE₂ pathways. Epidemiological studies frequently report the highest colonization rates or strongest associations in Stage II-III CRC, supporting this progression-linked role.

Late-stage/post-therapy dynamics: In advanced or treated disease, factors like chemotherapy, altered gut architecture, and systemic inflammation can dramatically shift the microbial landscape, potentially reducing the detectable abundance or clinical relevance of specific commensals like *pks*^+^* E. coli*, which may explain reports of inverse correlations in late-stage cohorts.

#### Geographical heterogeneity and appraisal of epidemiological robustness

The epidemiological strength of the *pks*^+^* E. coli*-CRC link varies markedly by region, underscoring the influence of cohort-specific factors:

Strong positive associations: Studies in France [58] and Malaysia [53] report high prevalence (55–74%) and significant odds ratios linking mucosal *pks*^+^* E. coli* to CRC, forming a cornerstone of the epidemiological evidence.

Nuanced or weak associations: Research from Japan [59] and some North American cohorts often finds more modest or non-significant differences in carriage rates between CRC patients and healthy controls.

Inverse correlations: Isolated reports, such as a Chinese cohort study [60], have observed an inverse relationship, with higher E. coli abundance in adenomas than carcinomas. This could reflect successful immune-mediated clearance of the bacterium during malignant transformation, competitive exclusion by other pathobionts in advanced tumors, or methodological artifacts related to sample type.

#### Towards a systematic assessment: meta-analytic perspectives and confounding factors

A rigorous, quantitative synthesis of this conflicting evidence is currently hampered by methodological inconsistencies across studies, which include: Sample Type & Site: Fecal samples vs. tumor-adjacent mucosa vs. intratumoral tissue yield different microbial profiles. Detection Methodology: Variability in PCR targets, sequencing platforms, and bioinformatic pipelines affects sensitivity and specificity. Population Characteristics: Differences in age, diet, antibiotic exposure, and comorbidities are rarely fully adjusted for.Tumor Characteristics: Studies often do not stratify by CRC molecular subtypes, which may interact differentially with the microbiome.

In conclusion, the statement that “*pks*^+^* E. coli* has varying effects at different stages” is supported by a synthesis of mechanistic and clinical data. The bacterium is a potent genotoxic initiator with a clear mechanistic signature, but its epidemiological footprint is modulated by tumor stage, geographical ecology, host factors, and study design. Therefore, it is more accurate to view *pks*^+^* E. coli* not as a universal CRC “driver” but as a context-dependent pathobiont whose clinical significance must be interpreted within a specific tumor microenvironment and patient phenotype. Future research should prioritize standardized, longitudinal cohort studies that integrate metagenomics with detailed host metadata to define the precise conditions under which *pks*^+^* E. coli* transitions from a commensal to a carcinogenic agent.

### Other intestinal microbiota associated with CRC

Beyond the core CRC-associated pathobionts discussed above, accumulating evidence has identified additional microbial species that may influence colorectal carcinogenesis through protective or detrimental mechanisms. The roles of these species are often more context-dependent and require further validation, but they underscore the complexity of host-microbe interactions in CRC.

*Streptococcus thermophilus*: This bacterium, commonly found in fermented dairy products, has been inversely correlated with CRC risk. Its potential protective role is primarily attributed to the secretion of β-galactosidase. Beyond safeguarding intestinal epithelial integrity, this enzyme has been shown to inhibit tumor cell proliferation in vitro. Mechanistically, β-galactosidase activity is associated with the suppression of oncogenic pathways in CRC cells, including the Hippo signaling pathway and the Warburg metabolic phenotype, which collectively curb cellular proliferation and viability. These anti-neoplastic effects have been corroborated in preclinical animal models of CRC. Furthermore, the presence of *S. thermophilus* is linked to an increased abundance of beneficial genera such as *Bifidobacterium* and *Lactobacillus*, suggesting its role may extend to promoting a broader health-associated microbial community [61, 62].

*Lactobacillus paracasei*: As a well-studied probiotic, L. paracasei exhibits multifaceted anti-tumor properties. The bacterium can specifically adhere to CRC cells and induce programmed cell death through the downregulation of the anti-apoptotic Bcl-2 protein family, a process potentially mediated via the PDK1/AKT/Bcl-2 signaling axis. Cell wall-associated proteins and other structural components have been identified as direct bioactive molecules that inhibit tumor growth [63, 64]. Expanding on these mechanisms, specific strains demonstrate unique properties: strain CMU-Pb-L5 can modulate polyamine metabolism in vivo, reducing protumorigenic polyamine levels to suppress tumor growth [65]; and postbiotic derivatives from strain CECT 9610 can inhibit store-operated calcium entry by downregulating Orai1/STIM1 expression, thereby impeding cancer cell migration [66]. Concurrently, strains such as *L. paracasei* DTA81 exert immunomodulatory effects by suppressing key pro-inflammatory mediators like IL-6 and IL-17, thereby disrupting the inflammatory cascade critical for early tumorigenesis [67].

A recent review by Agnes et al. explored the association between Streptococcus species producing gallic acid and CRC, highlighting the potential for these bacteria to increase CRC risk via the driver-passenger model [68]. The study also emphasized that exposure to Streptococcus gallic acid antigens correlates with a higher prevalence of CRC [69, 70]. However, the precise mechanisms underlying these associations remain to be elucidated. Table 1 provides a summary of gut microbiota species linked to CRC, alongside their proposed mechanisms of action.

**Table 1:** Overview of CRC-related microbiota characteristics

Bacterium	Experimental/epidemiological evidence	Proposed mechanism	References
*Peptostreptococcus anaerobius*	The detection rate in feces is positively correlated with the CRC stage	drives CRC via a PCWBR2-integrin α_2_/β_1_-PI3K-Akt-NF- κB signalling axis	[132]
*Enterococcus faecalis*	Inflammatory signaling pathways are increased in CRC cases colonized by *Enterococcus faecalis*	Activation of WNT/β-Catenin signaling pathways; directly damages host DNA via superoxide production	[133]
*Fusobacterium nucleatum*	Elevated levels detected in fecal samples and tumor tissues of CRC patients; promotes tumor progression in preclinical models.	Relies on virulence factors FadA and Fap2; associatedwith altered miRNA expression; recruits immunosuppressive myeloid cells.	[134]
*Enterotoxigenic Bacteroides fragilis*	The frequency of *Enterotoxigenic Bacteroides fragilis* among CRC and healthy patient was 58.3 and 26.6%	Stimulates inflammation via IL-17 and IL-8 production; activates STAT3 and TLR4 signaling pathways via Bft toxin.	[135]
Colibactin-producing *Escherichia coli*	Colibactin-producing *Escherichia coli* detected in 55.3% of CRC patient biopsies, compared to 19.3% in diverticulosis patient biopsies	Activation of inflammatory signaling cascades, Alter the tumor microenvironment	[136]

CRC, colorectal cancer; PCWBR2, putative cell wall binding repeat 2; PI3K, phosphoinositide 3-kinase; Akt, protein kinase B; NF-κB, nuclear factor kappa-light-chain-enhancer of activated B cells; IL, interleukin; TLR4, Toll-like receptor 4; Bft, *Bacteroides fragilis* toxin; FadA, Fusobacterium adhesion A; Fap2, outer-membrane adhesin

### Critical synthesis and clinical implications of key pathobionts

Synthesizing the evidence for Fn, ETBF, and CoPEC reveals distinct yet overlapping profiles that inform clinical translation.

Fn exhibits the strongest and most consistent association with advanced CRC stages, metastasis, and therapy resistance. Its mechanisms are particularly sophisticated, involving direct adhesion (FadA, Fap2), immune evasion (TIGIT engagement), and miRNA-mediated reprogramming. This makes Fn a prime candidate as a prognostic biomarker and a target for precision antimicrobial or anti-adhesion therapies, especially in chemotherapy-resistant or metastatic disease.

ETBF is strongly implicated in the early phases of carcinogenesis, driving chronic inflammation via IL-17/IL-8 and STAT3 activation. Its detection, especially in precancerous lesions, positions it as a promising risk-stratification and early-detection biomarker. Therapeutic strategies could focus on toxin neutralization, biofilm disruption, or targeted antibiotics to intercept the adenoma-carcinoma sequence.

CoPEC presents the most complex and heterogeneous picture. Evidence for its role is highly stage-dependent and variable across cohorts, with links to both initiation and progression. This heterogeneity limits its current utility as a standalone biomarker but underscores the importance of context—including tumor stage, geography, and host genetics—when evaluating its significance. Targeting CoPEC may be most relevant in specific high-risk populations or in combination with DNA repair-targeting agents.

Collectively, the distinct oncogenic profiles of these pathobionts underscore the necessity of moving beyond a universal microbiome-based strategy toward personalized, context-dependent interventions. Future strategies must integrate microbial profiling with tumor molecular subtypes and host factors to identify which patients harbor actionable microbial targets. This synthesis is visually summarized in Table 2, which maps the strength and consistency of evidence for each bacterium across clinical outcomes.

**Table 2:** Evidence strength and consistency map for CRC-associated microbiota

Outcome	* Fusobacterium nucleatum *		Enterotoxigenic* B. fragilis*		Colibactin-producing* E.coli*		References	
Tumor Initiation	Strong (The mechanism has been repeatedly validated in cells, animals, and human tissues)		Moderate (Research on the mechanism of animal models)		Strong (The core mechanism is clear and has been widely validated)		[137, 138]	
Tumor Progression	Strong (Clear immune escape mechanism)		Moderate (In depth research on pathway mechanisms)		Moderate(epidemiological association with stage II-III CRC in some studies, but less consistent than for initiation.)		[139, 140]	
Metastasis	Moderate (Indirect human clinical evidence)		Limited		Limited		[141]	
Therapy Response	Moderate (Research hotspots in translational medicine)		Preliminary evidence (Cell experiments and clinical observations)		Moderate (Novel mechanism and significant clinical significance)		[142, 143]	

## Intestinal microbiota in colorectal cancer treatment

Conventional therapeutic modalities for colorectal cancer, including adjuvant radiotherapy, chemotherapy, and immunotherapy, have markedly improved clinical outcomes and survival rates. Nevertheless, these interventions frequently induce significant alterations in gut microbial composition and function, creating a state of dysbiosis that may paradoxically promote therapeutic resistance and facilitate disease recurrence through complex host-microbe interactions and microenvironmental remodeling [69–71]. Consequently, comprehensive investigation of gut microbiome dynamics in the context of CRC therapeutics represents a critical research frontier, with the potential to identify novel microbial biomarkers and therapeutic targets. Such insights could revolutionize treatment paradigms by enabling microbiome-based precision medicine approaches that optimize therapeutic efficacy, minimize adverse effects, and ultimately improve long-term patient outcomes through targeted modulation of tumor-microbiome interactions.

### The role of intestinal microbiota in radiotherapy

Current therapeutic strategies for CRC demonstrate stage-dependent efficacy, with surgical resection achieving favorable outcomes in early-stage disease. However, advanced CRC presents significant clinical challenges, necessitating multimodal therapeutic approaches that integrate surgical intervention with systemic chemotherapy and radiation therapy. Radiotherapy has emerged as an essential component of the therapeutic arsenal for locally advanced CRC, providing synergistic effects with surgical resection [72]. Notably, emerging evidence suggests that the gut microbiome plays a crucial modulatory role in radiotherapy response, potentially influencing both treatment efficacy and toxicity through complex host-microbe interactions [73, 74].

Pelvic chemoradiotherapy induces significant alterations in gut microbial ecology, characterized by decreased microbial diversity and depletion of beneficial taxa, including *Enterococcus faecalis* and *Firmicutes* species. Interestingly, specific microbial populations, particularly within the *Firmicutes* and *Bacteroidetes phyla*, demonstrate immunomodulatory properties that may potentiate radiation-induced anti-tumor immunity [75, 76]. Emerging evidence suggests that targeted probiotic interventions can optimize radiotherapy outcomes. Their efficacy is highly strain-specific. Clinically researched strains, primarily from the genera *Lactobacillus* (e.g., *L. rhamnosus GG*) and *Bifidobacterium*, are often selected for their validated abilities to modulate intestinal barrier function, mitigate inflammation, and reduce treatment-related toxicities like diarrhea [77]. However, translating this potential into universal clinical benefit remains challenging. Prophylactic use does not yield consistent efficacy, as therapeutic response heavily depends on the host’s baseline microbial features [78]. Therefore, a precision approach is essential. Future strategies should utilize pretreatment metagenomic profiling to identify patients with specific dysbiotic signatures (e.g., depletion of short-chain fatty acid producers) who are most likely to benefit from tailored probiotic regimens containing defined, clinically validated strains.

Another key microbial metabolite, Butyrate, a product of microbial metabolism, can exert its anti-cancer effects through the modulation of immune mechanisms [79]. Specifically, butyrate inhibits histone deacetylase (HDAC), an enzyme critical to cell cycle regulation and growth. Recent studies have demonstrated that *butyrate* enhances radiotherapy sensitivity by inhibiting HDAC and modulating the activity of the transcription factor FOXO3A, a key regulator of cell growth and the cell cycle. Moreover, *butyrate* can further amplify radiotherapy efficacy by boosting the anti-tumor immune response [80–82].

The relationship between the microbial metabolite butyrate and cancer therapy is complex and context-dependent, a phenomenon known as the “butyrate paradox” [83]. This paradox is fundamentally defined by a strict dose-dependence, which has been consistently demonstrated in vitro. For instance, in human HCT-116 colorectal cancer cells, treatment with sodium *butyrate* at varying concentrations (0–20 mM) induced a dose-dependent response: most pro-apoptotic markers (e.g., BAX, CASP3, PUMA; *p* < 0.001) increased, while cell proliferation indicators like Ki-67 significantly decreased from concentrations of 10 mM onwards [84]. This illustrates the sharp transition from potential pro-proliferative effects at lower millimolar concentrations (e.g., 0.5–2 mM) to pro-apoptotic and anti-proliferative effects at higher concentrations (≥ 5–10 mM).In vivo, the situation is further complicated by tissue-specific responses and timing. Studies in animal models and ex vivo human tissue show that butyrate’s effect on key oncogenic pathways can vary. For example, a physiologically relevant dose (10 mM) can suppress the expression of the pro-tumorigenic gene osteopontin (OPN) in tumor colon tissue, while a lower dose (2 mM) was shown to upregulate OPN expression in a mouse colon epithelial cell line (MCE301) [85]. This discrepancy underscores that butyrate’s impact is not only concentration-sensitive but also critically dependent on the cellular and microenvironmental context, including the tissue type (normal, adenoma, carcinoma) and the baseline transcriptional state.

The efficacy of both probiotic and butyrate-based microbiome interventions in radiotherapy is critically context-dependent. The “butyrate paradox” and the strain-specificity of probiotics highlight that universal application is ineffective. Success therefore hinges on precision patient selection. Future strategies must integrate pretreatment profiling to match specific interventions—whether defined probiotic strains or dose-calibrated butyrate modulation—to an individual’s unique microbial and metabolic landscape.

### The role of intestinal microbiota in chemotherapy

The intestinal microbiome influences chemotherapeutic response through multifaceted mechanisms, including immunomodulation of anti-tumor immunity, biotransformation of therapeutic agents, dynamic microbial community restructuring, development of therapeutic resistance pathways, and production of bioactive microbial metabolites that modulate tumor biology and treatment efficacy [86]. The therapeutic activity of conventional chemotherapeutic agents, such as 5-fluorouracil(5-FU), cyclophosphamide (CTX), gemcitabine, and oxaliplatin, is significantly influenced by microbial-mediated processes, including enzymatic drug activation, immune potentiation, and metabolic modulation, which collectively determine treatment response and clinical outcomes [87–89].

CTX exerts unique immunomodulatory effects through intricate interactions with the gut microbiome, including translocation of specific Gram-positive bacteria to lymphoid tissues, modulation of intratumoral T cell dynamics favoring cytotoxic over regulatory populations, and functional reprogramming of immunosuppressive Treg cells. These microbiota-dependent mechanisms collectively enhance anti-tumor immunity and potentiate chemotherapeutic efficacy. Microbial metabolism significantly influences CTX pharmacology through bioactive metabolite production. Notably, gut microbiota-mediated *tryptophan* catabolism generates *indole derivatives* that serve as *aryl hydrocarbon receptor* ligands, potentiating intratumoral immune activation and enhancing therapeutic response through immunometabolic regulation [90].

*Sodium butyrate*, a metabolite derived from the intestinal microbiota, exerts anti-cancer effects by promoting cellular apoptosis. The combination of sodium butyrate with 5-FU or *oxaliplatin* demonstrates synergistic anti-tumor effects, significantly enhancing suppression of malignant phenotypes including proliferation, migration, and invasion while promoting apoptotic cell death. This therapeutic synergy not only improves treatment efficacy but also reduces chemotherapy-associated toxicity, offering a promising strategy for optimizing CRC management [91, 92].

While exerting potent anti-neoplastic effects, conventional chemotherapeutic agents significantly disrupt gut microbial ecology, causing decreased diversity and substantial shifts in microbial population dynamics. These treatment-induced alterations in microbiome composition may paradoxically influence therapeutic outcomes and contribute to treatment-related complications.5-FU administration induces significant microbial dysbiosis, characterized by selective enrichment of *Proteobacteria* and concomitant depletion of *Firmicutes* and *Actinobacteria* populations. This treatment-induced microbial imbalance may compromise chemotherapeutic efficacy through multiple mechanisms, including impaired drug metabolism and promotion of resistance pathways, highlighting the critical role of microbiome homeostasis in maintaining treatment responsiveness.

The selective enrichment of specific bacterial taxa, particularly *Sutterella* and *Veillonella dispar*, in chemotherapy-treated patients implicates these microorganisms in the development of therapeutic resistance. This microbial signature suggests potential mechanisms of treatment failure and highlights the need for microbiome-targeted strategies to overcome chemoresistance in cancer therapy [93]. The frequent detection of Fn enrichment in tumor tissues from chemotherapy-resistant CRC patients, particularly those experiencing disease recurrence, suggests a potential mechanistic role in treatment failure. Preclinical studies indicate that Fn contributes to chemoresistance in CRC by targeting TLR4/MYD88 innate immune signaling and specific microRNAs, which subsequently activates the autophagy pathway and alters chemotherapeutic response [94]. This distinct microbial profile positions Fn as a promising predictive biomarker for both chemotherapeutic efficacy and long-term clinical outcomes, potentially enabling more personalized treatment strategies in CRC management.

Yuan et al. [95] provided mechanistic insights into the microbiota-chemotherapy interaction using a colorectal cancer murine model. Their findings demonstrated superior tumor regression in 5-fluorouracil-treated mice compared to antibiotic-pretreated animals, establishing a direct correlation between antibiotic-induced microbial dysbiosis and diminished chemotherapeutic response, thereby underscoring the critical role of intact gut microbiota in maintaining treatment efficacy.

Microbial metabolic activities represent a significant contributor to chemoresistance mechanisms in cancer therapy. Targeted modulation of microbiota-derived metabolic pathways offers a promising strategy to overcome therapeutic resistance and enhance chemotherapeutic efficacy. Future investigations should prioritize the development of microbiome-based interventions that optimize treatment outcomes while minimizing toxicity through precise regulation of microbial metabolic networks and their downstream effects on tumor biology.

The optimal timing of microbiota interventions relative to chemotherapy cycles is a critical determinant of their efficacy. In current clinical practice for colorectal cancer, a triphasic strategy is emerging: interventions are initiated 1–2 weeks before chemotherapy to “prime” a favorable microbial state, continued concurrently to “protect” the gut ecosystem during treatment, and intensified (e.g., via FMT) during treatment breaks or upon signs of resistance to “reset” the microbiota. To translate this into robust, generalizable therapy, future research must systematically compare the efficacy of prophylactic (pre-chemotherapy), concurrent (adjuvant), and salvage (post-resistance) modulation strategies. Resolving this question of timing is paramount for designing definitive clinical trials and maximizing therapeutic benefit.

### The role of intestinal microbiota in immunotherapy

Tumor immunotherapy has emerged as a promising strategy for cancer treatment by harnessing the body’s immune system to elicit anti-tumor responses. Current approaches to immunotherapy include immune checkpoint inhibitors (ICIs), therapeutic monoclonal antibodies, immune vaccines, and adoptive cell therapies [96, 97]. A growing body of evidence highlights the interplay between host immunity and the gut microbiota in modulating the efficacy of immunotherapy. This interaction can influence key ICIs—such as anti-programmed cell death protein-1 (PD-1), anti-programmed death ligand 1 (PD-L1), and anti-cytotoxic T lymphocyte-associated antigen 4 (CTLA-4)—thereby affecting the progression of CRC [98].

Emerging evidence identifies specific gut microbiota constituents, including ETBF, Fn, and *Lactobacillus rhamnosus GG*(LGG), as critical determinants of immunotherapy responsiveness in colorectal cancer. These microbial modulators influence treatment efficacy through complex interactions with host immune pathways, highlighting the potential for microbiome-based strategies to optimize immunotherapeutic outcomes [99–101]. Mechanistic studies reveal that Fn may enhance PD-L1 inhibitor efficacy through STING pathway activation, leading to increased *interferon-γ* production and subsequent anti-tumor immune activation. This microbial-mediated immunomodulation represents a promising avenue for improving checkpoint inhibitor therapy in colorectal cancer [102]. Ishikawa et al. [98] provided compelling evidence for microbiota-dependent immunotherapy response, demonstrating that germ-free and antibiotic-treated mice failed to respond to CTLA-4 blockade, while ETBF colonization restored therapeutic efficacy. These findings establish a critical role for specific microbial communities in mediating checkpoint inhibitor responsiveness.This restoration was linked to a Th1 immune response induced by ETBF in the lymph nodes, which subsequently enhanced endritic cell maturation within the tumor microenvironment (TME).

**Fig. 3:**
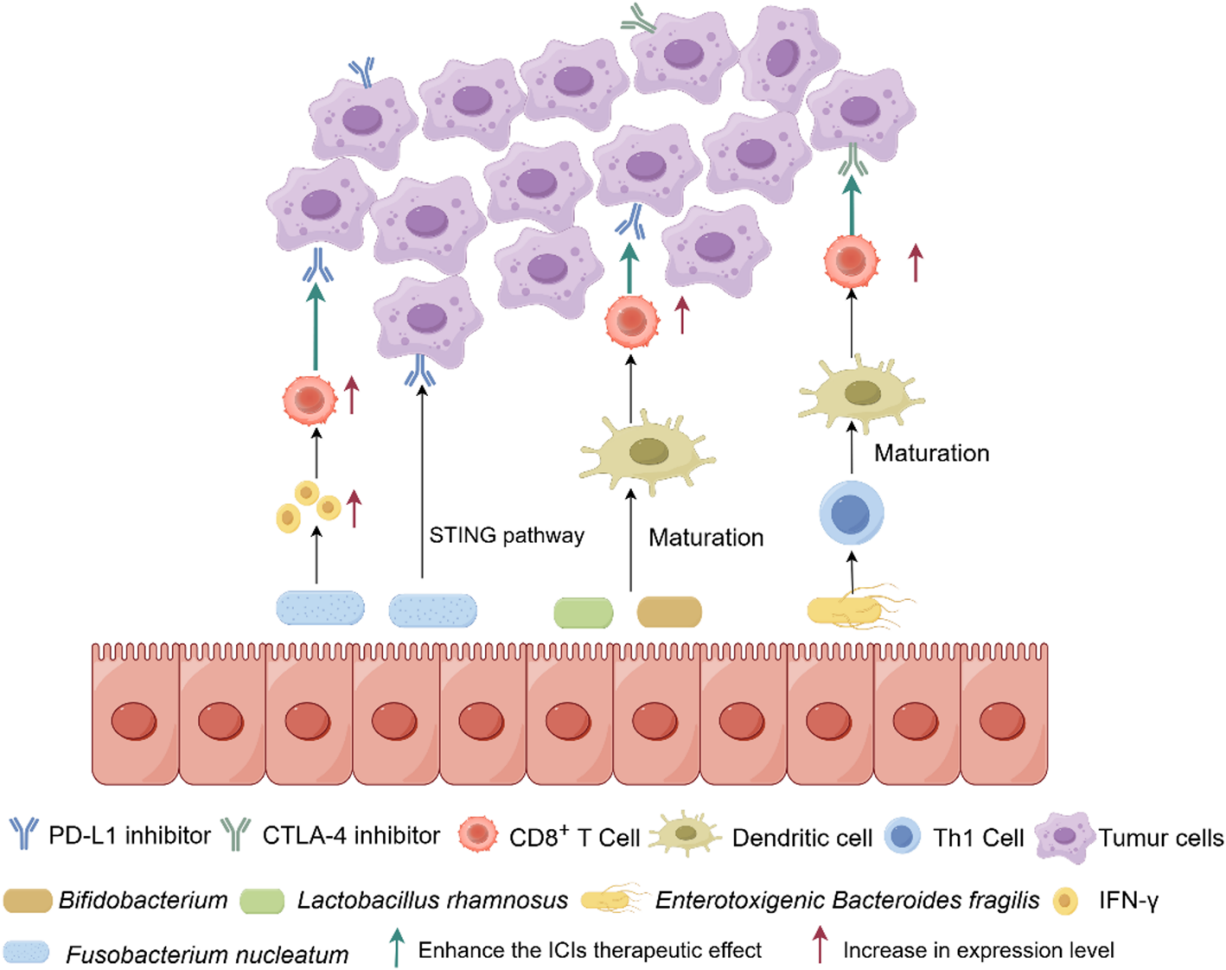
This figure illustrates the role of gut microbiota in modulating the response to immunotherapy. It highlights specific bacteria that augment the efficacy of immune checkpoint inhibitors, such as *Fusobacterium nucleatum*, *Bifidobacterium*, *Enterotoxigenic Bacteroides fragilis* and *Lactobacillus rhamnosus*. Additionally, this figure depicts interactions between immune cells (e.g., dendritic cells and CD8 + T cells) and key cytokines (e.g., IFN-γ). Figures were made with Figdraw

Specific Bifidobacterium species, particularly *B. brevis* and *B. longum* and LGG have been shown to potentiate anti-tumor immunity through dendritic cell activation, leading to enhanced CD8^+^ T cell priming and tumor infiltration. This microbial-mediated immunostimulation represents a promising strategy for improving T cell-mediated tumor control in the tumor microenvironment [103]. These findings suggest that targeted microbial interventions, through selective introduction of immunomodulatory bacterial strains, could enhance ICIs efficacy, representing a novel paradigm for optimizing cancer immunotherapy through microbiome engineering (Fig. 3).

ICIs mediate their anti-tumor activity by reprogramming the complex interplay between T lymphocytes, antigen-presenting cells, and malignant cells, effectively reversing tumor-induced immune suppression. This therapeutic approach has shown particularly promising outcomes in CRC patients exhibiting mismatch repair deficiency or high microsatellite instability (MSI-H), highlighting the importance of tumor immunogenicity in determining treatment response [104, 105]. However, the therapeutic potential of ICIs remains limited in the majority of CRC cases characterized by proficient mismatch repair and microsatellite stability(MSS).

The limited response of MSS CRC to PD-1/PD-L1 blockade is closely linked to an immunosuppressive tumor microenvironment, with the interleukin-17 A (IL-17 A) pathway playing a pivotal role. Elevated IL-17 A in MSS tumors promotes cancer cell survival and upregulates PD-L1 expression, creating a primary resistance mechanism to checkpoint inhibition [106].

Intriguingly, the gut pathobiont Fn appears to engage this resistance axis in a context-dependent manner. While chronic Fn infection can be pro-tumorigenic, emerging evidence suggests that in the MSS setting, Fn may activate the host STING pathway, leading to interferon-γ (IFN-γ) production and T cell activation [102, 107]. This immunostimulatory signal potentially counteracts the IL-17 A-mediated immunosuppression, thereby sensitizing tumors to PD-1 blockade.

These insights reveal a new therapeutic logic for MSS CRC: targeting the IL-17 A pathway or modulating specific microbiota like Fn could dismantle key resistance barriers. This supports the exploration of rational combination therapies, such as PD-1 inhibitors coupled with IL-17 A antagonists or precision antimicrobials against Fn, to unlock immunotherapy efficacy in this currently resistant population.

Targeted inhibition of IL-17 A signaling markedly potentiates PD-1 blockade efficacy in MSS CRC preclinical models, demonstrating the potential of combinatorial immunotherapeutic approaches to overcome treatment resistance.

Recent groundbreaking research by Yu Jun’s team has elucidated a novel mechanism by which Fn enhances PD-1 blockade responsiveness in MSS CRC [107]. Their findings demonstrate that fecal microbiota transplantation from Fn-high MSS CRC patients into murine models significantly augments anti-PD-1 therapeutic efficacy, providing compelling evidence for microbiome-mediated modulation of immunotherapy response. Moreover, single-dose Fn administration in MSS CRC allograft models and humanized mouse systems demonstrated comparable enhancement of PD-1 blockade efficacy, establishing Fn as a promising immunomodulatory agent.

## Microbiota-targeted intervention strategy

Building upon the intricate crosstalk between gut microbiota and CRC therapeutics, emerging microbiome-targeted interventions now offer promising avenues to modulate this dynamic interaction. These strategies aim to reprogram the tumor microenvironment through precision manipulation of microbial communities, with three principal approaches showing clinical relevance.

### Antibiotic

The impact of antibiotics on cancer therapeutics exhibits complex duality, shaped by factors such as antibiotic spectrum, timing of administration, microbial specificity, and the experimental or clinical context. Beneficial effects are often observed when antibiotics are applied selectively against specific, functionally relevant intratumoral bacteria. For instance, in *Fusobacterium*-positive patient-derived xenograft models of CRC, the nitroimidazole antibiotic metronidazole demonstrates a clear and tumor-specific benefit. It selectively depletes intratumoral *Fusobacterium*, leading to inhibited tumor growth, reduced bacterial load, and decreased tumor cell proliferation. Critically, this effect is exclusive to *Fusobacterium*-positive tumors, with no impact on negative ones, highlighting a microbiota-dependent mechanism [108]. Similarly, in a genetically predisposed murine model (5-week-old male ApcMin/+ mice), dietary administration of erythromycin (500 ppm in chow for 15 weeks) significantly reduced proximal intestinal polyp burden to 70.9% of untreated controls. This chemopreventive effect was associated with downregulation of pro-inflammatory mRNAs (IL-6, COX-2) in polyps, suggesting suppression of tumorigenesis through local anti-inflammatory activity [109]. Furthermore, in mice with multiple intestinal tumors colonized with ETBF, treatment with cefoxitin completely and durably eradicated ETBF colonization when administered 5 and 14 days post-inoculation. This clearance correlated with reduced mucosal IL-17 A expression and decreased tumorigenesis, underscoring the therapeutic potential of targeted bacterial eradication [110].

Conversely, broad-spectrum antibiotics can disrupt the commensal microbiota in a non-selective manner, which may impair the efficacy of certain cancer therapies, particularly immunotherapy [111] This is contrasted by agents like polyketide antibiotic, Mithramycin-A (Mit-A), which, despite its classification, exerts anti-tumor effects in immunocompetent murine models (e.g., the MC38 syngeneic orthotopic CRC model) primarily through direct immunomodulation of the tumor microenvironment rather than antimicrobial activity. When combined with anti-PD-L1 therapy in a therapeutic regimen beginning on day 6 and administered every other day until day 21, Mit-A enhanced T-cell infiltration, reduced immunosuppressive cells, and synergistically inhibited tumor growth [112]. This underscores that not all “antibiotics” function via microbiota depletion. In contrast, clinical meta-analyses suggest that broad-spectrum antibiotic-induced dysbiosis prior to or during immune checkpoint inhibitor therapy is frequently associated with diminished clinical response [113], highlighting the critical importance of timing, spectrum, and microbial context.

This duality underscores that indiscriminate antibiotic prophylaxis is inadvisable. Future strategies must evolve toward precision antimicrobial therapy, guided by the following principles: (1) Strict Indication: Clear separation between use for active infection/peri-operative prophylaxis and investigational use as adjuvant therapy; (2) Pathogen-Targeting: Prioritizing narrow-spectrum agents against documented oncopathobionts; and (3) Diagnostic Guidance: Incorporating microbial diagnostics to identify patients who may benefit from targeted intervention, thereby minimizing ecological disruption. Implementing this framework is crucial to harnessing the potential benefits of antibiotics while mitigating their risks in CRC management.

### Fecal microbial transplantation

Beyond conventional antimicrobial interventions, fecal microbial transplantation (FMT) has emerged as a promising strategy for gut microbiome regulation. This innovative procedure entails the introduction of viable microbial communities from donor stool into the recipient’s gastrointestinal tract to reestablish ecological homeostasis and elicit beneficial clinical outcomes [114].

Some preclinical evidence supports the role of FMT in modulating immunotherapy. In murine colorectal carcinoma models, FMT has been shown to reshape intestinal microbial profiles during programmed cell death protein-1 (PD-1) blockade, elevating populations of *ETBF* while reducing *Bacteroides ovale*, thereby enhancing the responsiveness to immune checkpoint inhibitors [115]. Notably, in an AOM/DSS-induced CRC mouse model, the combination of FMT (administered via enema) with intraperitoneally injected salinomycin not only inhibited tumor stem cells and restored microbial diversity but also improved the tumor immune microenvironment by increasing CD8^+^ T cell infiltration, suggesting a potential synergy in enhancing immunotherapy efficacy [116].

This therapeutic potential is beginning to translate into clinical observations. A recent clinical trial investigating the combination of FMT with anti-PD-1 therapy (NCT04130763) demonstrated that FMT capsules from healthy donors were well-tolerated and enhanced anti-PD-1 efficacy. The regimen involved FMT administration alone for three consecutive days, followed by combined treatment for six cycles. Analysis revealed that FMT increased gut microbial α-diversity and boosted IFN-γ^+^ CD8^+^ T cells in patients [117].

Collectively, these findings indicate that FMT holds promise for improving therapeutic outcomes in CRC by mitigating treatment-related toxicities and enhancing the antitumor effects of immune checkpoint inhibitors [118, 119]. Although FMT offers promising new avenues for the treatment of CRC, its application remains primarily in exploratory and early-phase clinical trial stages (see Table 3). Key considerations include rigorous donor screening—which has evolved from basic safety checks toward selecting “functional donors” based on microbial signatures (e.g., high diversity, abundance of beneficial taxa like SCFA producers)—and close monitoring for safety, including the risk of pathogen transmission. Thus, further investigation into the mechanisms by which FMT influences CRC is crucial, as it could provide valuable insights and open new directions for therapeutic strategies in CRC management.

**Table 3:** Landscape of selected clinical trials targeting the microbiome in colorectal cancer

Study and trial ID	Intervention and phase	Population	Key outcomes	Safety profile	Responder stratification
RENMIN-215 [144]	FMT + Tislelizumab (PD-1) + Fruquintinib Phase II, single-arm	Refractory MSS/pMMR mCRC (*n* = 20)	ORR: 20.0% mOS: 13.7months mPFS: Primary endpoint	Tolerable. 35% had grade 3 TRAEs.	MSS-specific
NCT04208958 [145]	Vancomycin + VE800(11-strain consortium) + Nivolumab Phase I	Anti-PD-1-refractory cancers, incl. MSS-CRC (*n* = 14)	ORR: 0% DCR: 21.4%	Well-tolerated	MSS-specific
NCT03775850 [146] NCT04729322 [147]	EDP1503(capsulecontaining Bifidobacterium animalis lactis) + Pembrolizumab Phase I/II, Open-label FMT+ Pembrolizumab Phase II, open-label, single-arm	Advanced/metastatic MSS Colorectal Cancer (*n* = 33), previously treated with standard chemotherapy MSI-H has multiple advanced cancers and is primarily resistant to PD-1 inhibitors (*n* = 15)	ORR:0%, no objective responses observed. ORR: 20%, Including1 case of complete remission	Manageable safety profile withno new safety signals Well-tolerated	MSS-specific MSI-H-specific

FMT, fecal microbiota transplantation; PD-1, programmed cell death protein 1; MSS, microsatellite stable; MSI-H, microsatellite instability-high. pMMR, proficient mismatch repair; mCRC, metastatic colorectal cancer; ORR, objective response rate; DCR, disease control rate; mOS, median overall survival; mPFS, median progression-free survival; TRAEs, treatment-related adverse events;

### Probiotics and intestinal microbiota

As research into the role of the gut microbiota in CRC progression advances, restoring microbial homeostasis has emerged as a crucial therapeutic strategy. Interventions targeting the microbiome encompass a spectrum of approaches: probiotics (live beneficial microbes), prebiotics (substrates selectively utilized by host microbes), synbiotics (combinations of probiotics and prebiotics), and postbiotics (bioactive microbial metabolites or inactivated cells). These strategies aim to correct gut microbiota imbalances and have shown promise in benefiting CRC patients [120].

Probiotics: Probiotics play a crucial role in modulating the body’s immune response. They can inhibit inflammatory processes in the intestinal mucosa, stimulate innate immune pathways, and enhance overall immune activity, thereby exerting potential antitumor effects. For instance, certain probiotics, such as Lactobacillus plantarum, promote the maturation of dendritic cells, which drives Th1 polarization, CD8⁺ T-cell activation, and natural killer cell migration, collectively enhancing anti-tumor immunity [121]. Clinical studies support this; tissue analysis from CRC patients has shown that specific probiotic regimens can reduce the abundance of pathogens like Fn while increasing beneficial butyrate-producing bacteria [122].

#### Critical considerations for clinical application

Strain selection: The primary probiotic genera include *Bifidobacterium*, *Lactobacillus*, and *Clostridium*. The core of selection lies in prioritizing clinically validated, specific strains tailored to CRC intervention needs (e.g., adjuvant therapy, side-effect alleviation) rather than generic species. Individual patient factors and formulation quality must also be considered.

Dosing reference: Preclinical models provide valuable dosing insights. For example, in ApcMin/+ mice and AOM/DSS-induced colorectal tumor models, Clostridium butyricum administered via gavage at a dose of 10⁸ CFU twice weekly demonstrated significant tumor suppression [123], offering a foundational reference for clinical dose determination.

Treatment duration: The optimal duration should be dynamically adjusted based on the treatment phase and patient recovery. For most postoperative CRC patients without contraindications, supplementation can be initiated on postoperative day 1 and continued for 7–14 days. When combined with immunotherapy, a pre-intervention and extended duration is often necessary to ensure the sustainability of the “microbiota-immune” synergistic effect.

Prebiotics: Prebiotics are a category of nutrients that help maintain gut microbial homeostasis and mitigate dysbiosis, potentially aiding in the prevention of inflammation and CRC [124]. These substances function by selectively stimulating the growth of beneficial bacteria, such as *Bifidobacterium* and *Lactobacillus*, while simultaneously suppressing the proliferation of oncogenic bacteria like ETBF, thereby modulating microbial balance.

Beyond this direct modulation, prebiotics exert multifaceted protective effects. They can reduce the concentration of harmful metabolites (e.g., ammonia) in the gut lumen, thereby limiting mucosal damage. Furthermore, by shaping a healthier microbiota, they promote goblet cells to secrete mucin-2, which is crucial for maintaining the integrity and stability of the colonic bilayer mucus barrier. Collectively, these actions work in concert to counteract the effects of dysbiosis and create a microenvironment less conducive to carcinogenesis.

Synbiotics: Synbiotics, defined as combinations of probiotics and prebiotics, leverage synergistic interactions and have shown positive effects in CRC management. CRC surgery often disrupts the gut microbiota, leading to complications such as infections and diarrhea. Synbiotics can modulate the microbial community to accelerate postoperative recovery. Meta-analyses indicate that synbiotic supplementation is effective in preventing postoperative infections and related complications in CRC patients, with the therapeutic effect appearing consistent regardless of probiotic strain formulation or the timing of administration [125].

Beyond the perioperative setting, synbiotics exhibit immunomodulatory potential relevant to carcinogenesis. They have been shown to increase IFN-γ production in CRC patients while preventing the aberrant elevation of IL-2 secretion in peripheral blood mononuclear cells from patients with resected intestinal polyps [126]. This ability to modulate the balance of inflammatory cytokines positions synbiotics as a potential strategy for suppressing tumor-associated inflammation.

 Postbiotics :The category of postbiotics includes inactivated microbial cells, cell fragments, and key metabolites such as SCFAs—notably butyrate and propionate. SCFAs are prime examples of how microbial metabolites exert direct, multifaceted effects: they inhibit HDAC, promote immune cell differentiation, strengthen the epithelial barrier, and suppress angiogenesis [127].

Postbiotics offer significant translational advantages over live biotherapeutics. They eliminate concerns regarding bacterial viability, colonization, and potential safety risks associated with administering live organisms. This allows for the precise dosing of well-defined bioactive molecules, facilitates pharmaceutical quality control, and may provide a superior safety profile, positioning postbiotics as a promising frontier for the development of next-generation, microbiome-derived drugs.

In summary, interventions targeting the gut microbiota offer complementary strategies for CRC management. Future success hinges on transitioning from generic approaches to precision applications that leverage the distinct advantages of each modality.

### Consideration of host and environmental context

The oncogenic impact of gut microbes is not uniform but is significantly modulated by host and environmental factors. Key contextual determinants include:

 Diet: Dietary patterns consistently reshape the gut ecosystem. The Western-style diet provides a prime example: it enriches for pathobionts like *pks*^+^* E. coli*, and cohort studies show that its associated CRC risk is significantly amplified in individuals with high mucosal abundance of this bacterium, demonstrating a clear diet-microbe synergy [128]. Conversely, high-fiber diets generally promote a protective microbiota.

Tumor molecular phenotype: MSI-H tumors, which are typically more immunogenic, may interact differently with the microbiome compared to MSS tumors. For instance, the efficacy of ICIs in MSI-H CRC is well-established and may be less dependent on specific microbiome modulators [129].

Prior therapies: The timing and type of prior therapies are critical. Broad-spectrum antibiotic use before or during immunotherapy consistently diminishes efficacy, whereas targeted antibiotic use against specific pathobionts is being explored as an adjuvant [130]. Chemotherapy itself drastically alters the microbiota, creating a window for interventions to restore a therapeutic-friendly community.

Host genetics: Polymorphisms in immune-related genes can influence individual responses to microbial components and shape the CRC risk associated with dysbiosis [131]. Understanding these interactions is crucial for personalizing microbiome-targeted strategies. Future interventions will likely need to stratify patients based on a combination of microbial, tumor, and host factors to predict and optimize therapeutic benefit.

## Methodological considerations and future directions

To translate promising associations into causal understanding and effective therapies, several methodological challenges must be addressed:

Study design: Most evidence is from cross-sectional studies, which cannot establish causality or temporal dynamics. Future research requires longitudinal cohort studies tracking microbiome changes from precancerous stages through treatment.

Confounding factors: Diet, medications, age, and lifestyle are major confounders. Studies must rigorously collect and adjust for these variables.

Technical standardization: Heterogeneity in sample collection, DNA extraction, sequencing platforms, and bioinformatic pipelines hinders comparison across studies. Consensus on standardized protocols is urgently needed.

From correlation to mechanism: Metagenomic sequencing identifies “who is there” but not “what they are doing.” Integrating metatranscriptomics, metabolomics, and functional assays in gnotobiotic models is essential to delineate mechanistic pathways.

Personalized medicine approach: As summarized in Table 2, the evidence strength varies by pathogen and outcome. The field must move towards integrated models that combine microbial signatures with host genetics (e.g., MSI status) and clinical parameters to identify which patients will benefit from specific microbiome modulations.

Addressing these limitations will be critical for developing robust microbial biomarkers and moving microbiota-targeted therapies from promising concepts to standardized clinical practice.

## Conclusions and outlook

In recent years, scientific investigations have increasingly concentrated on exploring the correlation between gut microbiome composition and various aspects of colorectal cancer, including its development, disease progression, therapeutic interventions, and clinical outcomes, thereby establishing a scientific basis for implementing microbiome-targeted treatment strategies in CRC management. However, our understanding of the microbiome remains limited, and it remains challenging to determine whether a single microbial species or a complex consortium of microorganisms contributes to disease development, or whether interactions among microbiota play a significant role. Consequently, additional research efforts are necessary to comprehensively unravel the molecular pathways and biological processes through which gut microbial communities influence colorectal carcinogenesis.

The therapeutic potential of intestinal microbial communities in colorectal cancer management has emerged as a significant focus of contemporary oncology research. Beneficial microbial species not only appear to inhibit carcinogenesis but may also enhance the efficacy of radiotherapy, chemotherapy, and immunotherapy, offering new therapeutic avenues. Moreover, these microorganisms could represent potential targets for future CRC interventions. The complex etiology of colorectal cancer extends well beyond microbial influences, encompassing multiple external determinants including nutritional patterns, behavioral habits, environmental exposures, and hereditary factors that collectively shape the genomic landscape of malignant cells. Although scientific comprehension of the dynamic interactions between host microbiota and neoplastic genetic alterations is still at a preliminary stage, this emerging research domain presents substantial opportunities for advancing our knowledge of CRC development.

## Data Availability

No datasets were generated or analysed during the current study.
